# Ubiquitin Modification of SARS-CoV-2 Membrane Protein Promotes Virion Assembly and Budding via Autophagy

**DOI:** 10.1080/27694127.2022.2100040

**Published:** 2022-07-14

**Authors:** Zhen Yuan, Binbin Ding

**Affiliations:** a Department of Biochemistry and Molecular Biology, School of Basic Medicine, Tongji Medical College, Huazhong University of Science and Technology, Wuhan, Hubei, China; b Cell Architecture Research Institute, Huazhong University of Science and Technology, Wuhan, Hubei, China

**Keywords:** Autophagy, PSMD14, release, RNF5, SARS-CoV-2, ubiquitination, virus-like particles

## Abstract

In our recent study, we reported the molecular mechanisms of SARS-CoV-2 assembly and budding. Envelope protein (E) and membrane protein (M) of SARS-CoV-2 form complexes that ensure the uniform size of viral particles for viral maturation and budding. The E3 ligase RNF5 mediates the ubiquitination of M at residue K15 and thus enhances M-E interaction, whereas the deubiquitinating enzyme PSMD14/POH1 negatively regulates this process. Intriguingly, we show that M traffics from the Golgi apparatus to an LC3-positive phagophore and exploits the autophagosome to egress, and this process is dependent on RNF5-mediated ubiquitin modification of M and M-E interaction. Our finding suggests that RNF5 and PSMD14 play important roles in SARS-CoV-2 release and SARS-CoV-2-induced exploitation of autophagosomes for egress.

Virion release is an essential step in the release of the enveloped virus particles, and the process ultimately triggers the separation of virion and host membranes. Ubiquitin modification is important for this final process. Like other enveloped virus particles, those of SARS-CoV-2 are released from the membrane of infected cells and viral membrane protein (M) is necessary for this process. However, much remains unknown on the utilization of the ubiquitin system for assembly and release by SARS-CoV-2: 1) How M mediates virion budding; 2) whether ubiquitination is required for SARS-CoV-2 release, and which are the E3 ligase and the deubiquitinating enzyme that play crucial roles; 3) how the virion traffics to the plasma membrane for release, and which pathways or organelles can be used.

In our latest study [1], we used the convenient virus-like particles (VLPs) to screen and identify the key cellular proteins and pathways that function in viral release. VLPs are recombinant structures made up of one or more different viral molecules, mimicking the size and shape of viruses but lacking the genetic material, which are reportedly useful tools for studying the viral assembly and release processes of many enveloped viruses. Utilizing approaches combining VLP systems, transmission electron microscopy and the NanoSight NS300 system, we found that viral E with M are required and sufficient for SARS-CoV-2 VLP assembly and budding; the interaction of E with M ensures the uniform size distribution of VLP particles. E undergoes homo-oligomerization through its C-terminal domain. However, E_ΔCTD_ still promotes M-mediated viral release while an E_ΔNTD_ mutant, which fails to bind to M, loses its ability for promoting viral release, suggesting that E-M interaction, not E homo-oligomerization is essential for VLPs release.

To identify host factors essential for virion release, we performed a small-scale screening of RNAi targeting candidates that were on the list from SARS-CoV-2 M IP/MS. RNF5 and PSMD14/POH1 were identified as E3 ligases and deubiquitinating enzymes of M, respectively. Wild-type RNF5, but not its catalytic dead mutant RNF5^C42S^, promotes VLPs and extracellular viral production. Wild-type PSMD14, but not its catalytic dead mutant C120S, causes the reduction of VLPs and SARS-CoV-2 virus release. RNF5 ubiquitinates M at the residue K15 to enhance the interaction of M with E, thus enhancing the stability of the M-E complex on the membrane and ensuring the uniform size of VLPs to promote viral release. While PSMD14 acts as a deubiquitinating enzyme of M and negatively regulates virion release by inhibiting the interaction between M and E. Our data showed that the expression of PSMD14 was decreased while RNF5 was increased in SARS-CoV-2-infected cells compared to control cells, indicating that SARS-CoV-2 decreases the expression of PSMD14 and increases the expression of RNF5 to ensure the ubiquitin modification of M and viral release. It will be interesting to determine the mRNA or protein levels of RNF5 or PSMD14 in SARS-CoV-2-infected human tissues.

Macroautophagy/autophagy, a multistep process engulfing a portion of cytoplasmic components within a double-membrane autophagosome and delivering them to lysosomes for degradation, plays important roles in pathogen invasion. Viruses have evolved sophisticated strategies to evade autophagy and even utilize it for their own replication and survival. We then asked whether SARS-CoV-2 uses autophagic machinery for budding. We found that CQ or torin 1 treatment significantly enhance VLP release, and knockdown of ATG7 significantly decreases VLP release, suggesting a critical role of autophagy in virion release. M expression alone displays a significant overlap with the Golgi apparatus marker GOLGA2/GM130. Surprisingly, when co-expressed with E, M exhibits a notable colocalization with LC3. Our data also showed that M expression enhances autophagy flux and ORF3a blocks the degradation of SQSTM1/p62 induced by M expression, suggesting that in SARS-CoV-2-infected cells, M uses autophagosomes, not autolysosomes, for release. M fails to colocalize with LC3 in RNF5 KD cells, and its ubiquitin modification defect mutant K15R fails to target LC3-positive phagosomes, suggesting that RNF5 promotes the trafficking of M from the Golgi apparatus to phagosomes, a process which is dependent on its E3 ligase activity. Base on the emerging evidence, we hypothesized that SARS-CoV-2 uses autophagosomes to egress (Figure 1). Our unpublished data indicate that M does not establish interactions with autophagic proteins, suggesting that M induces autophagy via a new autophagic regulatory factor. Using siRNA screening, we identified a novel candidate that regulated autophagy and established interactions with M, which might serve as a key adaptor between autophagy and M.

**Figure 1.**
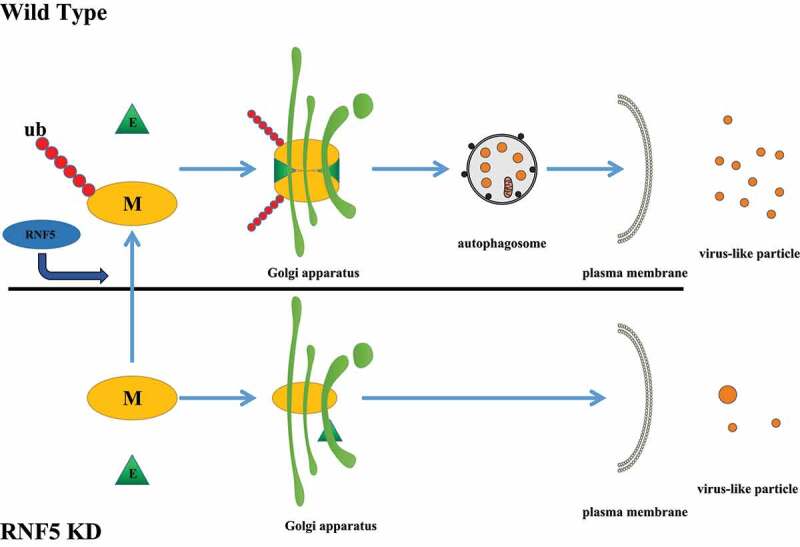
E3 ligase RNF5 enhances the interaction between viral M and E by mediating the ubiquitination of SARS-CoV-2 M. M-E complexes traffic from the Golgi apparatus to phagosomes, and use autophagosomes for viral release.

Our study showed the molecular mechanisms of SARS-CoV-2 M-mediated VLP release, and identified the E3 ligase RNF5 as mediating ubiquitin modification of SARS-CoV-2 M to stabilize the M-E complex and traffic to phagosomes for virion release. We think this work is a major advance in our understanding of the mechanism of SARS-CoV-2 release, and provides RNF5 as a potential target for antiviral drug development.

There are several limitations in this study: for obtaining most conclusions, we relied on VLP systems rather than wild-type viruses. As such, the real recombinant and native virus need be used to investigate SARS-CoV-2 assembly and release to complement our VLP system.
